# Author Correction: Modelling of free-form conformal metasurfaces

**DOI:** 10.1038/s41467-020-18924-5

**Published:** 2020-10-01

**Authors:** Kedi Wu, Philippe Coquet, Qi Jie Wang, Patrice Genevet

**Affiliations:** 1 CINTRA, UMI 3288, CNRS/NTU/Thales, Research Techno Plaza, 50 Nanyang Drive, Singapore, 637553 Singapore; 2 Center for OptoElectronics and Biophotonics (COEB), School of Electrical and Electronic Engineering, Nanyang Technological University, Singapore, 639798 Singapore; 3 Université Côte d’Azur, CNRS, CRHEA, rue Bernard Gregory, Sophia Antipolis, 06560 Valbonne, France; 4 Present Address: Department of Information Physics and Engineering, Nanjing University of Science and Technology, Nanjing, 210094 China

**Keywords:** Techniques and instrumentation, Applied optics, Metamaterials

Correction to: *Nature Communications* 10.1038/s41467-018-05579-6, published online: 28 August 2018.

The original version of this Article contained in Eq. (6). The last term of this equation incorrectly read “$E_x^{n - 1}\left( {n_x,n_j} \right)$”, instead of “$E_x^{n + 1}(n_x,n_j)$”.

The correct form of Eq. (6) is:

$$E_x^{n + 1}(n_x,n_j + 1) =	 \frac{{1 - j\omega \chi _{ee}^{ux}dt/4ds}}{{1 + j\omega \chi _{ee}^{ux}dt/4ds}}E_x^n\left( {n_x,n_j + 1} \right)\\ 	+ \frac{{dt}}{{\varepsilon _0ds(1 + j\omega \chi _{ee}^{ux}dt/4ds)}}\left( {H_z^{n + 1/2}\left( {n_x,n_j + 1} \right) - H_z^{n + 1/2}\left( {n_x,n_j} \right)} \right)\\ 	 - \frac{{j\omega \chi _{ee}^{ux}dt/4ds}}{{1 + j\omega \chi _{ee}^{ux}dt/4ds}}\left( {E_x^n\left( {n_x,n_j} \right) + E_x^{n + 1}\left( {n_x,n_j} \right)} \right).$$

This has been corrected in both the PDF and HTML versions of the Article.

The original version of the Supplementary Information associated with this Article contained an error in Supplementary Eq. (6). The last term of this equation incorrectly read “$E_y^{n - 1}(n_i,n_y)$”, instead of “$E_y^{n + 1}(n_i,n_y)$”.

The HTML has now been updated to include a corrected version of the Supplementary Information.

The corrected equations do not affect the simulations results and conclusions presented in the Article.

