# Supplementary material for: Modelling of free-form conformal metasurfaces
Source: Nat Commun. 2018 Aug 28;9:3494. doi: 10.1038/s41467-018-05579-6 (PMC6113266; doi:10.1038/s41467-018-05579-6)
Supplement: Supplementary file 1 — Supplementary Information [file 41467_2018_5579_MOESM1_ESM.pdf]

SUPPLEMENTARY INFORMATION

Modelling of Free-form Conformal Metasurfaces

Supplementary Note 1

Derivations of Modified FDTD Equations

In the following, we will describe the Finite-Difference Time-Domain method (FDTD) generalized sheet transition conditions (GSTC) theory. In conventional FDTD methods, metasurface is considered as a layer with a nonzero thickness ds, given by the spatial grid along x, y, and z directions. Considering that the metasurface is placed at $x = n_i ds$, the discretized field components are calculated with normal FDTD update equation everywhere except at the discontinuity, meaning that along the entire simulation space except at the (n_i th, n_j th) node, the fields are as follows,

$$\begin{aligned} H_z^{n+1/2}(n_x,n_y) &= H_z^{n-1/2}(n_x,n_y) \\ &+ \frac{dt}{\mu_0 ds}\left(E_x^n(n_x,n_y+1) - E_x^n(n_x,n_y) + E_y^n(n_x,n_y) - E_y^n(n_x+1,n_y)\right), \end{aligned} \tag{1a}$$

$$E_x^{n+1}(n_x,n_y) = E_x^n(n_x,n_y) + \frac{dt}{\varepsilon_0 ds}\left(H_z^{n+1/2}(n_x,n_y) - H_z^{n+1/2}(n_x,n_y-1)\right), \tag{1b}$$

$$E_y^{n+1}(n_x,n_y) = E_y^n(n_x,n_y) + \frac{dt}{\varepsilon_0 ds}\left(H_z^{n+1/2}(n_x-1,n_y) - H_z^{n+1/2}(n_x,n_y)\right), \tag{1c}$$

in which n_x, n_y are the discretized position coordinate along x, y directions, and dt, n are the time step and discretized time coordinate. At the metasurface ($n_x = n_i$), the fields on both sides are written following the metasurface synthesis [26-28],

$$-\Delta H_z^{n+1/2} = \mathrm{j}\omega\varepsilon_0 \chi_{\mathrm{ee}}^{yy} E_{y,\mathrm{av}}^{n+1/2}, \tag{2a}$$

$$-\Delta E_y^n = \mathrm{j}\omega\mu_0 \chi_{\mathrm{mm}}^{zz} H_{z,\mathrm{av}}^n, \tag{2b}$$

The subscript " av " denotes average of the fields on both sides of the metasurface, formally written as $E_{y,\mathrm{av}}^{n}=(E_{y}^{n}(n_i+1,n_y)+E_{y}^{n}(n_i,n_y))/2$, $E_{y,\mathrm{av}}^{n+1/2}=(E_{y,\mathrm{av}}^{n+1}+E_{y,\mathrm{av}}^{n})/2$, $H_{z,\mathrm{av}}^{n+1/2}=(H_{z}^{n+1/2}(n_i,n_y)+H_{z}^{n+1/2}(n_i+1,n_y))/2$, and $H_{z,\mathrm{av}}^{n}=(H_{z,\mathrm{av}}^{n+1/2}+H_{z,\mathrm{av}}^{n-1/2})/2$.

At this point we need to consider the updated equation for $H_{z}^{n+1/2}(n_i+1,n_y)$ at the metasurface since it cannot be directly obtained from Eq. (1) due to the discontinuity expressed in Eq. (2). To resolve this problem, it is necessary to introduce a virtual electric node $E_{y}^{n-1}(-,n_y)$ at the lower side of the metasurface and further substituted it into Eq. (1). $H_{z}^{n+1/2}(n_i+1,n_y)$ is thereafter given by the expression:

$$\begin{aligned}&H_{z}^{n+1/2}(n_i,n_y)=H_{z}^{n-1/2}(n_i,n_y)\\&+\frac{dt}{\mu_0 ds}(E_{x}^{n}(n_i,n_y+1)-E_{x}^{n}(n_i,n_y)+E_{y}^{n}(n_x,n_y)-E_{y}^{n}(-,n_y))\end{aligned}\tag{3}$$

Using the GSTC expression, as in Eq. (2), $E_{y}^{n}(-,n_y)$ can be calculated as

$$\begin{aligned}&E_{y}^{n}(-,n_y)=E_{y}^{n}(n_i,n_y)-\mathrm{j}\omega\mu_0\chi_{\mathrm{mm}}^{zz}H_{z,\mathrm{av}}^{n+1/2}\\&=E_{y}^{n}(n_i,n_y)-\mathrm{j}\omega\mu_0\chi_{\mathrm{mm}}^{zz}(H_{z}^{n+1/2}(n_i,n_y)+H_{z}^{n+1/2}(n_i+1,n_y)\\&+H_{z}^{n-1/2}(n_i,n_y)+H_{z}^{n-1/2}(n_i+1,n_y))/4.\end{aligned}\tag{4}$$

Substituting Eq. (4) into Eq. (3), we obtain:

$$H_z^{n+1/2}(n_i,n_y)=\frac{1-\mathrm{j}\omega\chi_{\mathrm{mm}}^{zz}dt/4ds}{1+\mathrm{j}\omega\chi_{\mathrm{mm}}^{zz}dt/4ds}H_z^{n-1/2}(n_i,n_y)$$
$$+\frac{dt}{\mu_0 ds(1+j\omega\chi_{\mathrm{mm}}^{zz}dt/4ds)}(E_x^n(n_i,n_y+1)-E_x^n(n_i,n_y)+E_y^n(n_i,n_y)-E_y^n(n_i+1,n_y)) \quad (5)$$
$$-\frac{\mathrm{j}\omega\chi_{\mathrm{mm}}^{zz}dt/4ds}{1+\mathrm{j}\omega\chi_{\mathrm{mm}}^{zz}dt/4ds}(H_z^{n+1/2}(n_i+1,n_y)+H_z^{n-1/2}(n_i+1,n_y)).$$

Note that the magnetic field across the metasurface, $H_z^{n+1/2}(n_i+1,n_y)$, can still be calculated using the normal FDTD update equation as in Eq. (1). This is because all field components used to update $H_z^{n+1/2}(n_i+1,n_y)$ are on one side of the metasurface and do not cross the discontinuity. Similarly, $E_y^{n+1}(n_i,n_y)$ is also calculated by Eq. (1c). By introducing a virtual magnetic node $H_z^{n-1/2}(+,n_y)$ at the upper side of the metasurface and following the same derivation procedure of $H_z^{n+1/2}(n_i,n_y)$, the update equation of $E_y^{n+1}(n_i+1,n_y)$ can be written as

$$E_y^{n+1}(n_i+1,n_y)=\frac{1-\mathrm{j}\omega\chi_{\mathrm{ee}}^{yy}dt/4ds}{1+\mathrm{j}\omega\chi_{\mathrm{ee}}^{yy}dt/4ds}E_y^n(n_i+1,n_y)$$
$$+\frac{dt}{\varepsilon_0 ds(1+\mathrm{j}\omega\chi_{\mathrm{ee}}^{yy}dt/4ds)}(H_z^{n+1/2}(n_i,n_y)-H_z^{n+1/2}(n_i+1,n_y)) \quad (6)$$
$$-\frac{\mathrm{j}\omega\chi_{\mathrm{ee}}^{yy}dt/4ds}{1+\mathrm{j}\omega\chi_{\mathrm{ee}}^{yy}dt/4ds}(E_y^n(n_i,n_y)+E_y^{n+1}(n_i,n_y)).$$

The electric components $E_y^{n+1}(n_i,n_y)$ around the metasurface can be obtained from Eq. (1).

The set of equations (1) - (4) have been implemented in a customized FDTD numerical software which calculates the evolution of field distributions around a metasurface following the FDTD discretization scheme presented in

Fig. 1. In between each time step, the fields originating from the source are updated for propagation by implementing the time-harmonic waves. Fields across the metasurfaces are obtained by substituting electric and magnetic susceptibility tensors χ_{ee} and χ_{mm} into Eqs. (5) and (6) as sketched in Figs. 1b and 1c.

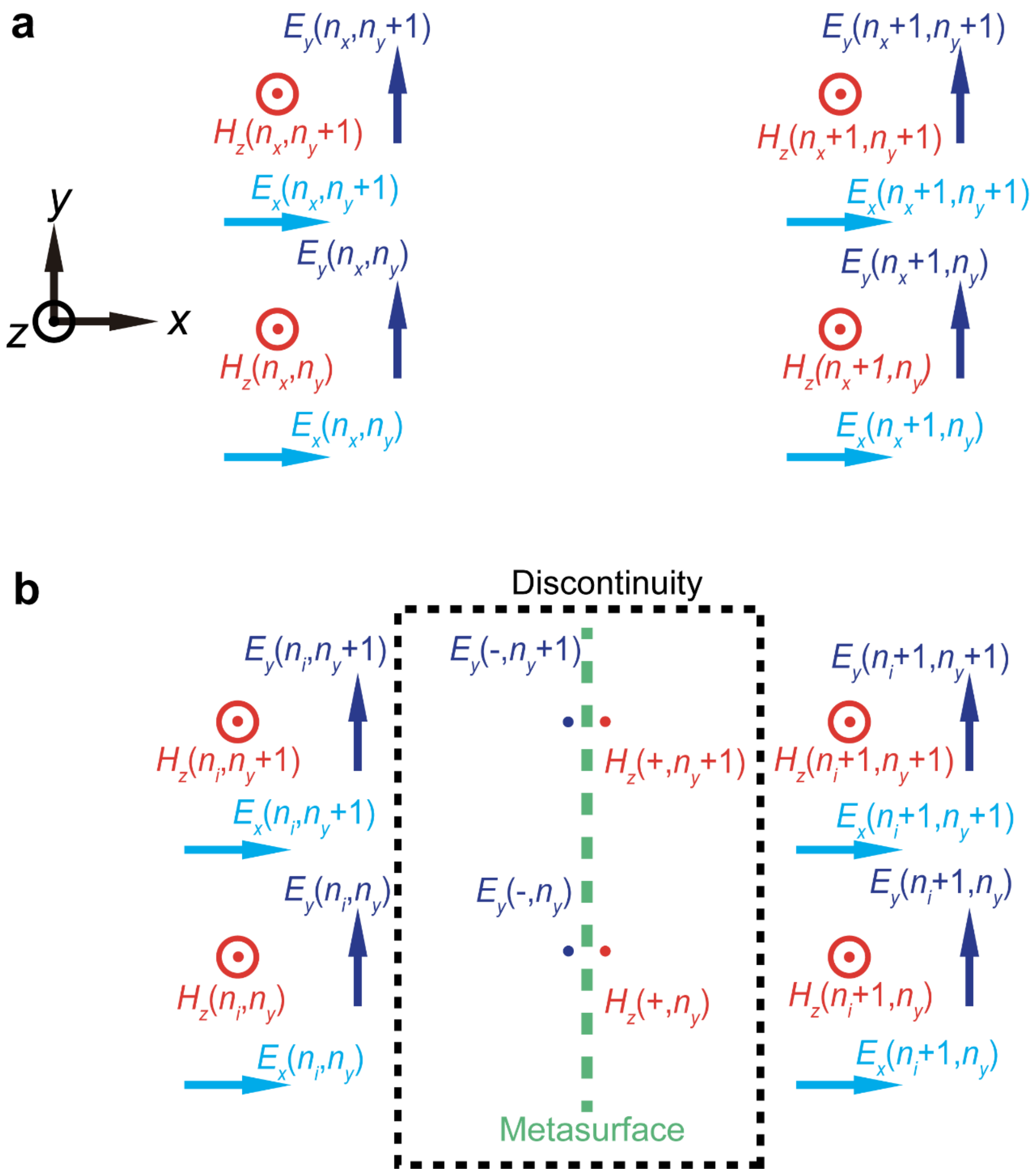

Fig. 1. The algorithm uses normal FDTD method to calculate the light field except at positions corresponding to the metasurfaces. (a) In the absence of metasurface layers, normal Cartesian Yee cells are used to calculate electric and magnetic field vector components. (b) At the planar metasurface, FDTD-GSTCs, as given by Eqs. (5), (6) are implemented by inserting virtual nodes around to the normal Yee cell. The modification includes introducing virtual

magnetic nodes $H_z^{n-1/2}(+,n_y)$ and virtual electric nodes $E_y^{n-1}(-,n_y)$ to calculate the field along the plane $x = n_i ds$.

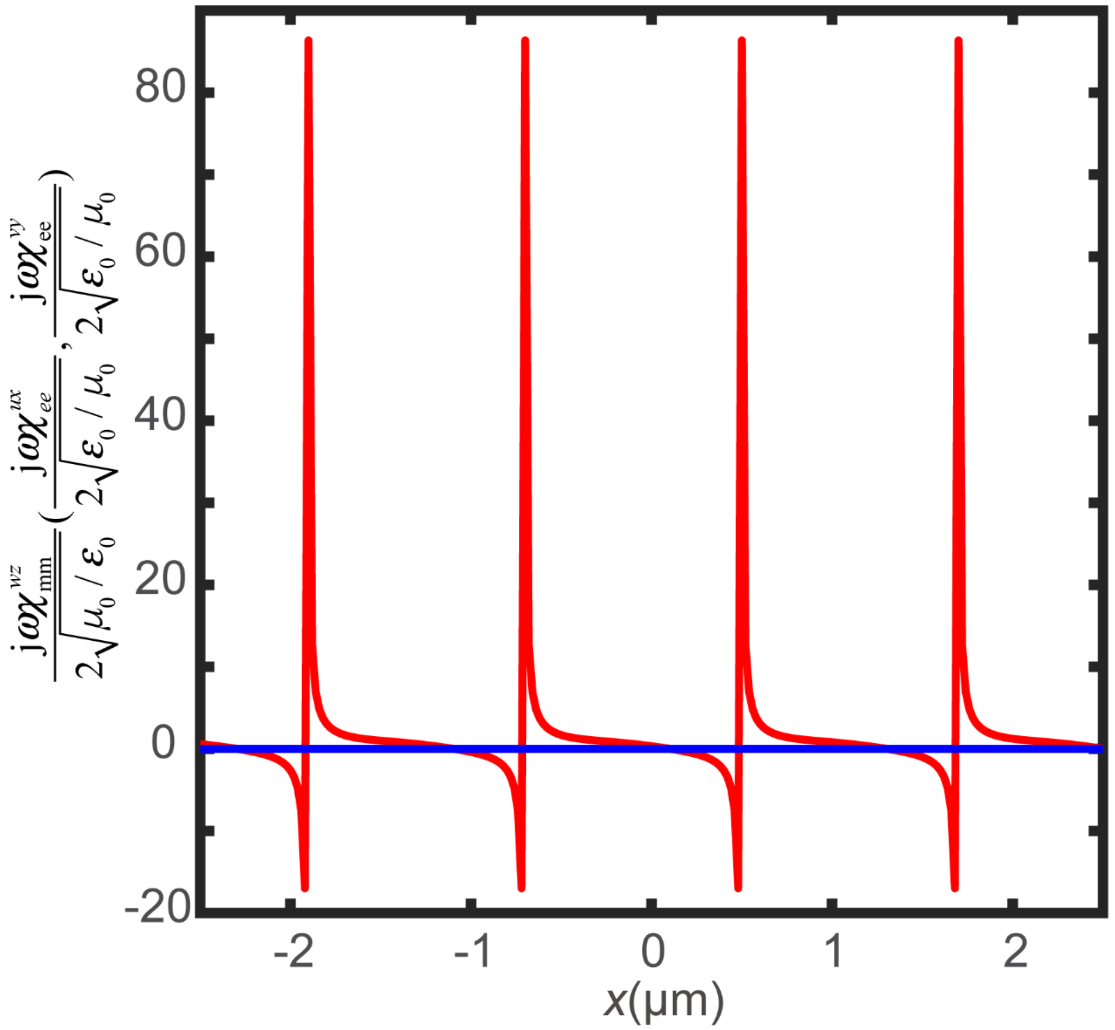

Fig. 2. The values of the susceptibilities of the curved beam deflector. Red (blue) curve represents the imaginary (real) part of the susceptibility tensor.

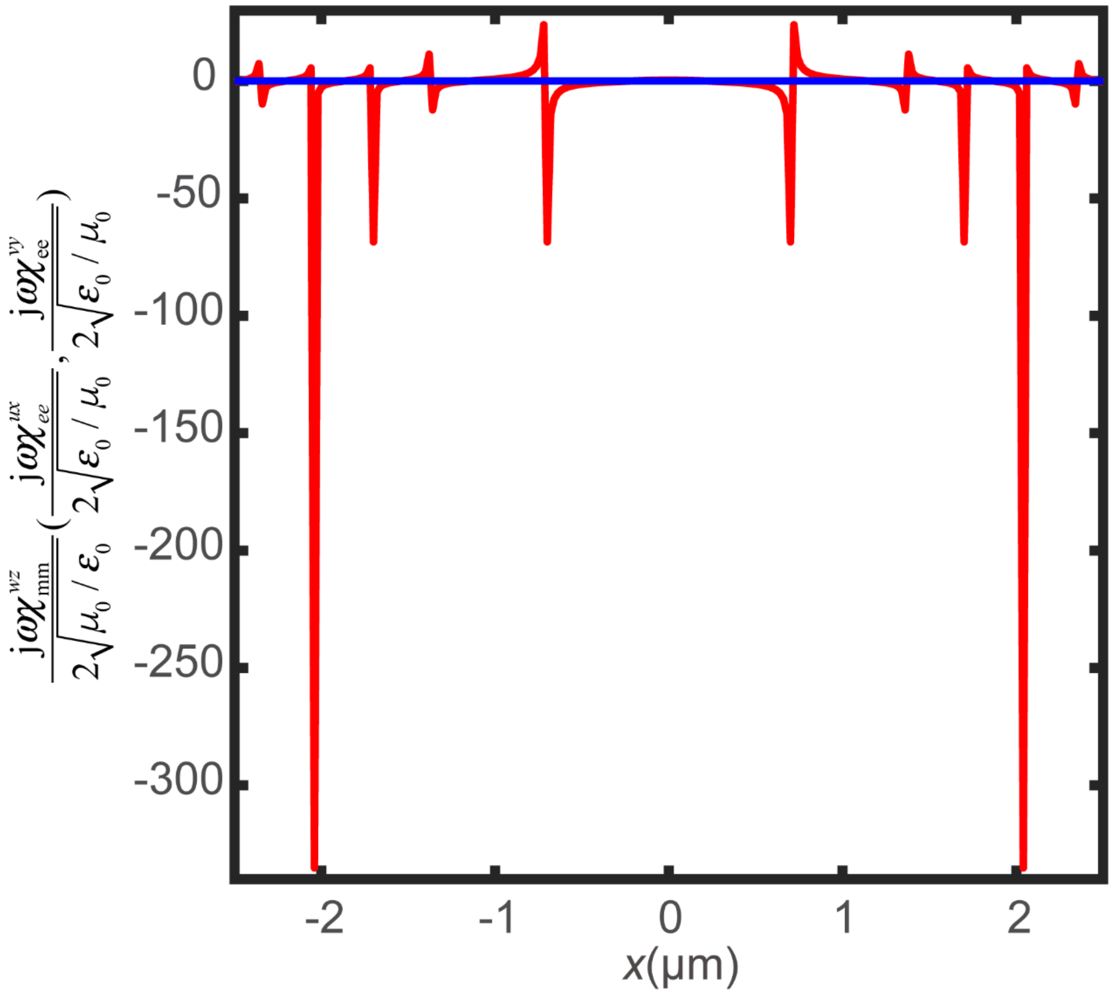

Fig. 3. The values of the susceptibilities of the curved lens. Red (blue) curve represents the imaginary (real) part of the susceptibility tensor.

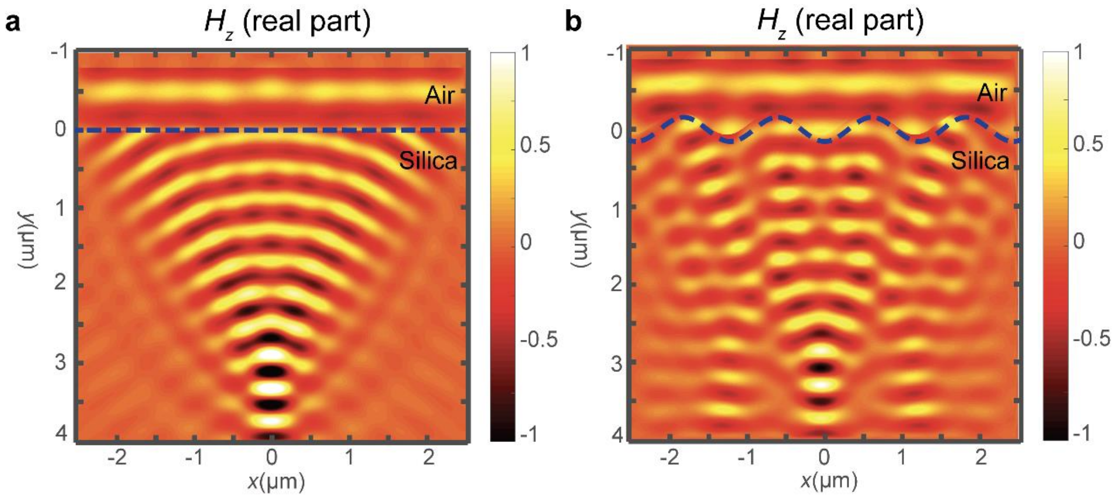

Fig. 4. From flat to curved meta-lenses at the interface of the air and silica. Magnetic field intensity distribution of light transmitted through a planar (a) and a free-form (b) lens placed at the interface of the air and silica substrate. In the calculations, the incident light is a TM polarized plane wave propagating along the y-direction. Blue dashed lines indicate the interfaces. These results show that conformal boundary optics are successfully implemented in a modified FDTD numerical scheme.
